# Förster resonance energy transfer: Role of diffusion of fluorophore orientation and separation in observed shifts of FRET efficiency

**DOI:** 10.1371/journal.pone.0177122

**Published:** 2017-05-19

**Authors:** Bram Wallace, Paul J. Atzberger

**Affiliations:** 1 Department of Mathematics, University of California Santa Barbara, Santa Barbara, CA, 93106, United States of America; 2 Department of Mechanical Engineering, University of California Santa Barbara, Santa Barbara, CA, 93106, United States of America; Consiglio Nazionale delle Ricerche, ITALY

## Abstract

Förster resonance energy transfer (FRET) is a widely used single-molecule technique for measuring nanoscale distances from changes in the non-radiative transfer of energy between donor and acceptor fluorophores. For macromolecules and complexes this observed transfer efficiency is used to infer changes in molecular conformation under differing experimental conditions. However, sometimes shifts are observed in the FRET efficiency even when there is strong experimental evidence that the molecular conformational state is unchanged. We investigate ways in which such discrepancies can arise from kinetic effects. We show that significant shifts can arise from the interplay between excitation kinetics, orientation diffusion of fluorophores, separation diffusion of fluorophores, and non-emitting quenching.

## 1 Introduction

Förster resonance energy transfer (FRET) is a widely used single-molecule technique for measuring distances within and between molecules [1, 2]. FRET is based on non-radiative transfer of energy between an excited donor molecule and an acceptor molecule. Förster developed theory for non-radiative transfer based on dipole-dipole interactions [1, 3]. For the separation distance *R*, Förster’s theory predicts an energy transfer efficiency scaling as ∼(*R*/*R*_0_)^−6^. In practice one typically has *R*_0_ ∼ 1nm [1–3]. Experimental realization using FRET as a “spectrocopic ruler” for distance measurements within single molecules was introduced in the experiments of Stryer and Haugland in the 1960’s [2, 4, 5]. Since this time, FRET has continued to be developed and has become a versatile tool widely used in the biological sciences and biotechnology [6–10].

In the biological sciences, FRET has been used to report on protein-protein interactions [11, 12]. At the single-molecule level, FRET has been used to measure distances between labels in characterizing the structures and dynamics of macromolecules including RNA, DNA, proteins, and their molecular complexes [6, 13–15]. Time-depend FRET measurements have been developed to characterize reaction kinetics of enzymes [6, 16–18], ligand-receptor interactions [7, 19–21], conformational dynamics of proteins [13, 22, 23], and movement of molecular motor proteins [24, 25].

Many types of molecules can be used for acceptor-donor pairing in FRET. Some molecules have photophysics that result in non-emitting quenching when interacting with surrounding chemical species or intramolecular chemical groups [26–30]. This provides ways for FRET probes to be used to report on the localized concentration of chemical species, such as metal ions [26, 31] in water or Ca^+^ ions released during neuronal activity [15]. In emerging biotechnology, FRET is also being used to develop new types of high-fidelity sensors for single-molecule detection and high-throughput assays for screening [7, 8, 20].

In single-pair FRET (spFRET), a single pair of acceptor and donor molecules are used to measure intramolecular distances [4]. To characterize different molecular conformational states or the heterogeneous states of subpopulations, a ratiometric analysis is used to estimate the transfer efficiency *E* [18, 32]. Over repeated measurements this is reported typically as a histogram of the efficiency values *E*. Under differing experimental conditions, such as introduction of a denaturant, shifts in the observed efficiency histogram are interpreted as changes in the molecular conformational state [6, 14, 23, 33]. In recent experiments by Lipman et al. [34, 35], it has been observed that in some situations such FRET shifts may occur even when there are no apparent changes in the conformational state. This finding is supported by experiments where x-ray scattering of molecules indicate no conformational change or the molecular structure involved is inherently rigid such as a polyproline chain [34, 35]. There is a precedent for such changes in efficiency occuring due to properties of the medium. Experiments such as those by Zhang, Fu, Lakowicz, and others [36, 37] demonstrate that the presence of foreign particles (specifically silver in their studies) can affect the donor-acceptor interaction. Furthermore, results by Makarov and Plaxco [38] suggest for a flexible polymer that not just the conformational state but the end-to-end kinetics can effect observed FRET efficiency.

This presents the important issue of characterizing how shifts can occur in FRET efficiency in the apparent absence of any change in the conformational state. We investigate using theory and stochastic simulations the roles played by excitation kinetics, orientation diffusion of fluorophores, separation diffusion of fluorophores, and non-emitting quenching. Our results aim to quantify the magnitude of these effects and to help identify regimes in which these factors could impact experimental measurements.

## 2 Förster resonance energy transfer (FRET)

### 2.1 Transfer efficiency

The FRET efficiency is the fraction of energy that is transferred non-radiatively from the donor to the acceptor molecule. Initially, it will be assumed that the energy can only be emitted as a donor photon or non-radiatively transferred to the acceptor ultimately to be emitted as an acceptor photon. In this case, the transfer efficiency is related to the rates *κ*_*A*_ and *κ*_*D*_ of the photon emission of the donor and acceptor as

$$\begin{array}{c} E=\frac{\kappa_{A}}{\kappa_{A}+\kappa_{D}} \end{array}$$ (1)

**Fig 1:**
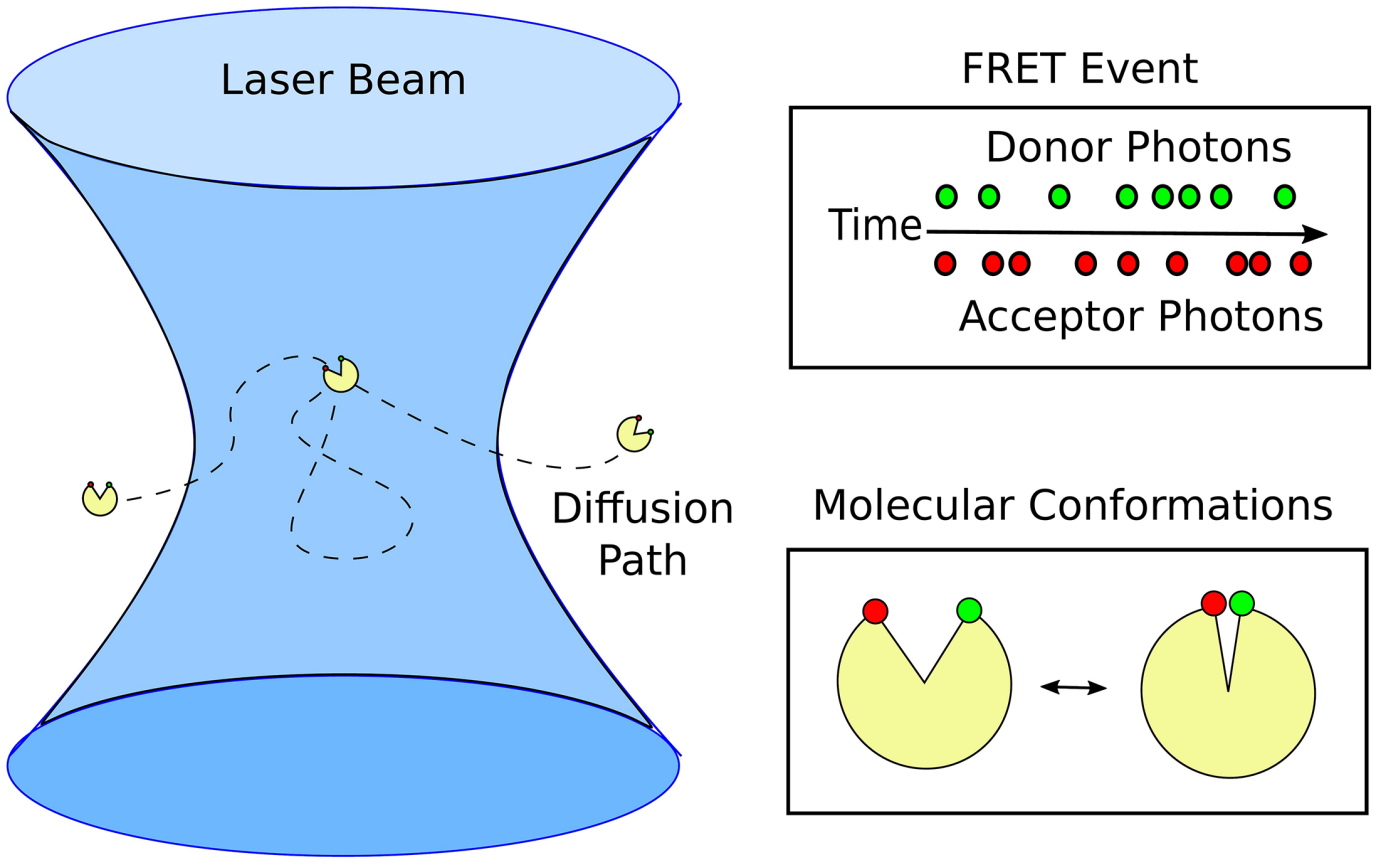
Förster resonance energy transfer (FRET). The donor molecule is excited to a higher energy state by an adsorbed photon. The donor relaxes back to its ground state either by emitting a photon or transferring energy to the acceptor molecule. The excited state of the acceptor molecule relaxes by emitting photons. Shown are the two widely used donor-acceptor dyes *Cy*3 and *Cy*5.

We illustrate the donor-acceptor transfer process in Fig 1.

For some systems it may be important to consider also additional photo-chemical states as in [39, 40] or transfer of energy from collisions with other molecules in solution that results in non-emitting quenching [28–30]. We consider some of these effects in subsequent sections.

Förster theory predicts that the non-radiative transfer rate *κ*_*T*_ depends on the donor-acceptor separation distance *R* as

$$\begin{array}{c} \kappa_{T}=\frac{1}{\tau_{D}}{\left(\frac{R}{R_{0}}\right)}^{-6} \end{array}$$ (2)

This is based on dipole-dipole interactions and separation distances smaller than the emitting photon wave-length [1–3]. The *τ*_*D*_ = 1/*κ*_*D*_ denotes the average lifetime of the excited state of the donor in the absence of the acceptor. The characteristic Förster distance *R*_0_ depends on the photophysics of the donor and acceptor molecules through

$$\begin{array}{c} R_{0}^{6}=\frac{9\left(\ln\left(10\right)\right)\kappa^{2}\Phi_{D}J}{128\pi^{5}n^{4}N_{A}} \end{array}$$ (3)

The *N*_*A*_ is Avogodros’ number, *κ*^2^ a factor associated with the donor-acceptor dipole-dipole relative orientations [5, 41], Φ_*D*_ is the quantum yield of the donor fluorescence in the abscence of the acceptor, *J* is the overlap integral associated with the adsorption spectrum of the donor and acceptor, *n* is the index of refraction. For a more detailed discussion see [1–3, 5, 10, 42].

When all transferred energy is immediately emitted as an acceptor photon, we have *κ*_*A*_ = *κ*_*T*_. The distance dependence of the FRET efficiency can then be expressed as

$$\begin{array}{c} E=\frac{1}{1+{\left(R/R_{0}\right)}^{6}} \end{array}$$ (4)

Förster theory has the important utility that the donor-acceptor separation distance *R* can be inferred from observations of *E*. To obtain *R*_0_ only requires in principle knowledge of a few properties of the photo-physics of the donor and acceptor molecules. This allows for FRET to be used as an effective nanoscale ruler for molecular systems [4, 23, 24, 27, 43].

### 2.2 Single-pair FRET for molecules diffusing in free solution

**Fig 2:**
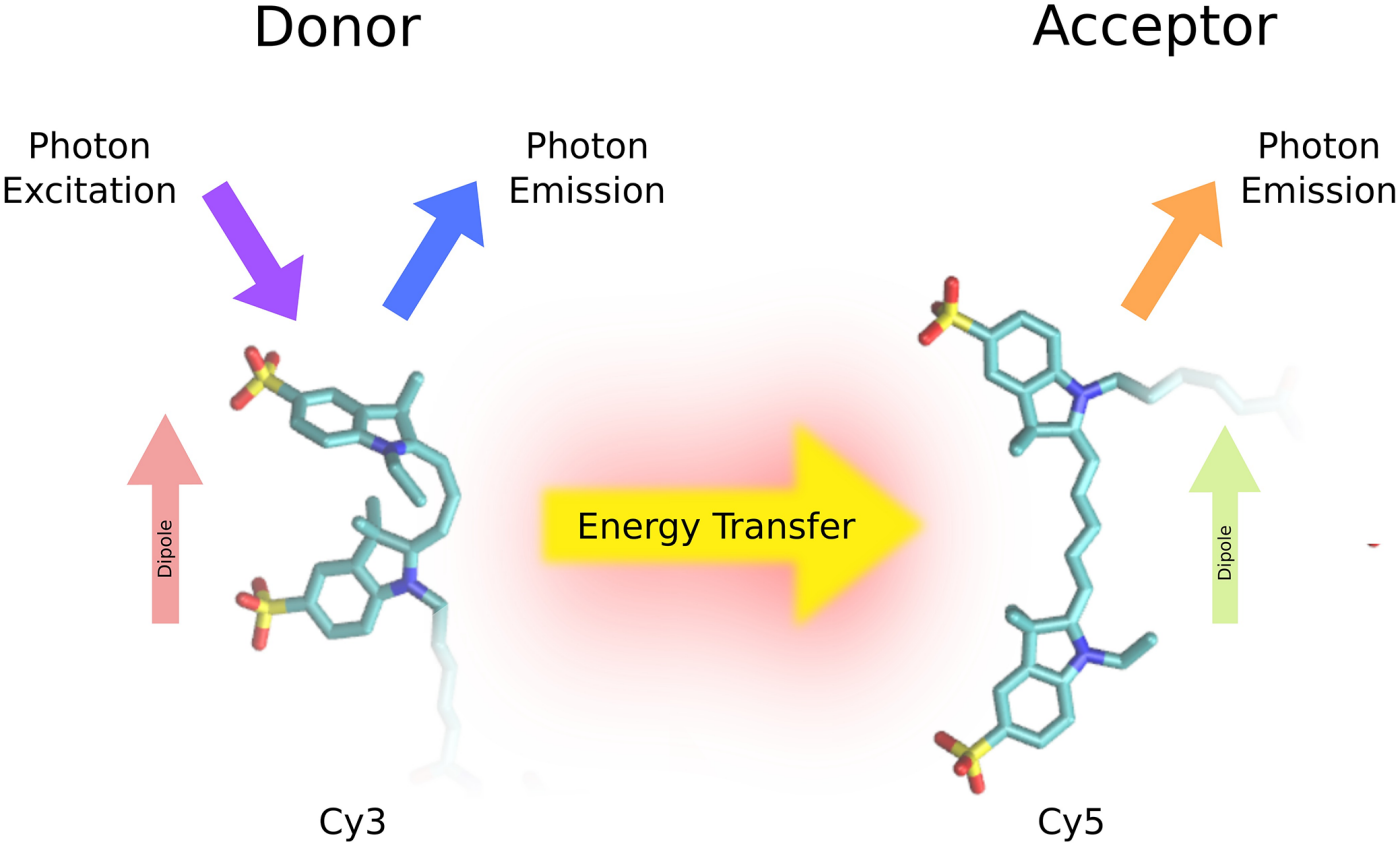
Single-molecule FRET event. A FRET event starts when a molecule labelled by a donor and acceptor pair diffuse into a volume of sufficiently large laser intensity near the focal point (left). The counts for detected photon emissions for the acceptor *n*_*A*_ and donor *n*_*D*_ are recorded until the molecule diffuses out of the focal volume (top right). During the donor excitation either a photon is emitted or energy is non-radiatively transferred to the acceptor and emitted with rates that depend on the molecular conformation (lower right).

To obtain single molecule measurements for freely diffusing molecules, the donor is typically excited by waiting for an individual molecule to diffuse into the focus of a laser beam [6, 18, 23, 24]. When the molecule is in a region near enough to the focal point of the laser (within the focal volume) the donor is excited with high probability and a sequence of donor and acceptor photon emissions occur, see Fig 2. During the time the molecule dwells in the focal volume, the number of detected donor and acceptor photons *n*_*D*_, *n*_*A*_ can be counted. This allows for a ratio-metric estimate of the transfer efficiency as [18, 32]

$$\begin{array}{c} E=\frac{n_{A}}{n_{A}+n_{D}} \end{array}$$ (5)

This experimental data for the FRET efficiency is then typically aggregated to form a histogram of the observed energy transfer efficiencies *E*. We remark that there are a number of important considerations in practice for such experiments, such as the development of criteria for when such a sequence of emissions is to be considered a significant FRET event or when there are short durations in the focal volume or shot noise.

The efficiency histogram provides a characterization of the relative proportions of different conformational states or sub-populations of the molecules encountered during a measurement. For the case of homogeneous molecules in the same conformational state, the efficiency histogram is expected to exhibit a narrow peak around the characteristic FRET efficiency corresponding to the donar-acceptor separation of the conformation. It is then natural to consider changes in the conformational state of the molecule by looking for shifts in the location of the peak in the FRET histogram. This is widely used in experimental practice to characterize biomolecular systems [6, 13, 14, 22].

However, in recent experiments by Lipman et al. [34, 35], it has been found that in some circumstances a significant shift can occur in the FRET efficiency histogram while there is no apparent change in conformational state. We use theory and stochastic simulations to investigate the roles played by kinetics. We initially investigate the role played by the rotational and translational diffusion of fluorophores on the time-scale of the excitation kinetics of the donor and acceptor molecules. We then consider the role of additional effects such as non-emitting quenching.

## 3 Importance of donor-acceptor kinetics

### 3.1 Donor-acceptor excitation and relaxation

We consider the role of the kinetics of donor and acceptor excitation, energy-transfer, and relaxation. We model the event of donor excitation as occurring at the rate *κ*_*D*_ = 1/*τ*_*D*_. The *τ*_*D*_ is the mean donor excitation life-time in the absence of the acceptor. A donor molecule in the excited state either relaxes by emitting a photon at the rate *κ*_*D*_ or by transferring energy to the acceptor molecule at the rate *κ*_*T*_ in accordance with Eq (2). We emphasize that in practice the rate *κ*_*T*_ depends on a number of factors. This includes the separation distance *R* between the donor and acceptor. This also depends on the relative orientations of the donor and acceptor which is captured by the *κ*^2^ term in Eq (3).

We investigate how such dependence of the energy transfer on the donor and acceptor configurations competes with the other excitation and relaxation kinetics. For this purpose we develop a stochastic model of the excitation-relaxation kinetics and perform simulations of the rotational and translational diffusion of the acceptor and donor molecules. We investigate the impact of these effects on the effective *κ*_*T*_ and observed FRET transfer efficiencies *E*.

### 3.2 Donor-acceptor orientation diffusion

The relative orientation of the dipole moments of the donor and acceptor molecules can significantly influence the efficiency of energy transfer [5, 41, 42, 44, 45]. This can be seen from the factor *κ*^2^ that contributes in Eq (3). The factor *κ* is given by [5, 41, 42]

$$\begin{array}{c} \kappa =\hat{d}\cdot \hat{a}-3\left(\hat{d}\cdot \hat{r}\right)\left(\hat{a}\cdot \hat{r}\right) \end{array}$$ (6)

The $\hat{a}$ and $\hat{d}$ denote the unit vectors representing the orientations of the dipole moments of the acceptor and donor molecules. The $\hat{r}$ gives the separation unit vector pointing from the donor to acceptor.

**Fig 3:**
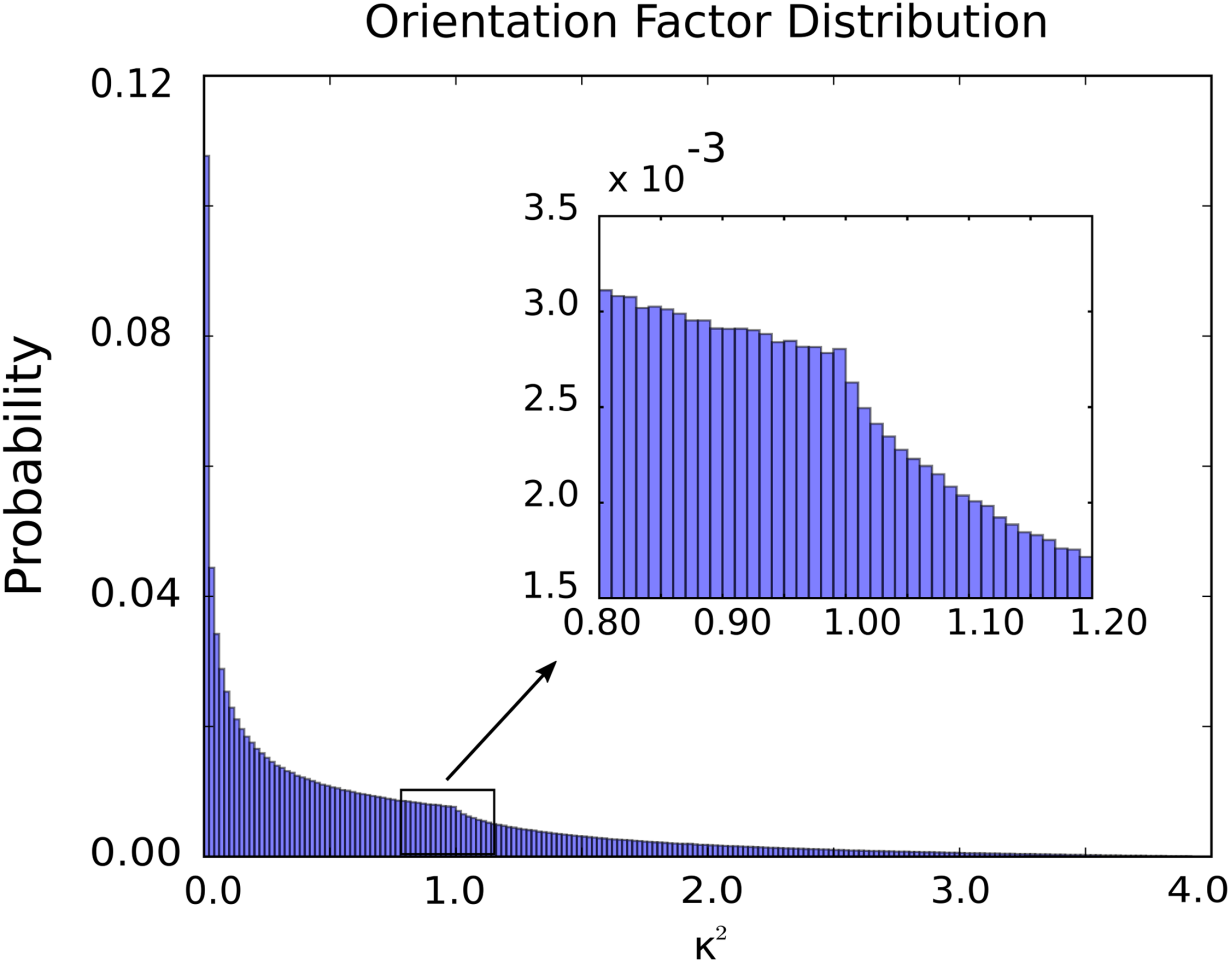
Distribution of the orientation factor *κ*^2^. Shown is the random acceptor-donor orientations for *κ*^2^ that is distributed between 0 and 4. The distribution exhibits a well-known cusp at *κ*^2^ = 1 (see inset). The majority of the distribution falls between *κ*^2^ = 0 and *κ*^2^ = 1 with a significant bias toward *κ*^2^ = 0. The histogram was constructed from 10^7^ random dye orientation pairs.

Contributions from orientation effects are often approximated by averaging assuming that the orientation rapidly diffuses isotropically on a time-scale much longer than the donor excitation time. The averaged orientation factor is often used 〈*κ*^2^〉 = 2/3, [41, 42]. However, in many situations the orientation diffusion can be comparable to the time-scale of excitations or from molecular-level sterics it may not be isotropic sampling all orientations [12, 41, 44, 46]. Also, even for rapid diffusion, experimental measurements often involve a small sample of the *κ*^2^ values which can range between 0 and 4. This is sampled from a distribution with irregular and asymmetric features, see Fig 3.

We investigate the role of orientation diffusion and its role on observed FRET efficiencies leading to possible shifts. Since only the relative angle between the donor and acceptor is relevant, we can model rotational diffusion by a Brownian motion on the surface of a sphere [47]. This can be expressed in spherical coordinates by the stochastic process

$$\begin{array}{ccc} \frac{d\Theta_{t}}{dt} & = & \frac{1}{2}\frac{D_{R}}{\rho^{2}}{\left(\tan\left(\Theta_{t}\right)\right)}^{-1}+\sqrt{\frac{D_{R}}{\rho^{2}}}\cdot \frac{dW_{t}^{\left(1\right)}}{dt}\\ \frac{d\Phi_{t}}{dt} & = & {\left(\sin\left(\Theta_{t}\right)\right)}^{-1}\sqrt{\frac{D_{R}}{\rho^{2}}}\cdot \frac{dW_{t}^{\left(2\right)}}{dt} \end{array}$$ (7)

The *D*_*R*_ denotes the diffusion coefficent on the surface and *ρ* the radius of the sphere. The equations are to be interpreted in the sense of Ito Calculus [48, 49]. The $W_{t}^{\left(1\right)}$ and $W_{t}^{\left(2\right)}$ denote independent Brownian motions. For a sphere of radius *ρ*, a configuration associated with the spherical coordinates (Θ_*t*_, Φ_*t*_) are to be interpreted in cartesean coordinates as *X*_*t*_ = *ρ* sin(Θ_*t*_)cos(Φ_*t*_), *Y*_*t*_ = *ρ* sin(Θ_*t*_)sin(Φ_*t*_), and *Z*_*t*_ = *ρ* cos(Θ_*t*_).

We perform simulations by numerically computing time-steps approximating the stochastic process in Eq (7). This is accomplished by projecting Brownian motion to the surface of the sphere. In particular, we use the time-stepping procedure

$$\begin{array}{ccc} {\tilde{w}}^{n+1} & = & w^{n}+\sqrt{D_{R}\Delta t}\eta_{3}^{n} \end{array}$$ (8)

$$\begin{array}{ccc} w^{n+1} & = & ({\tilde{w}}^{n+1}/∥{\tilde{w}}^{n+1}∥)\rho \end{array}$$ (9)

The $\eta_{3}^{n}$ is generated each step as a three-dimensional Gaussian random variable with independent components having mean zero and variance one. We remark this approach avoids complications associated with the spherical coordinates by avoiding the need to switch coordinate charts when configurations approach the degeneracies near the poles of the sphere [50].

We characterize the time-scale of the rotational diffusion by *τ*_*R*_ = 4*π*^2^*ρ*^2^/*D*_*R*_. We use for the dye length *ρ* = 1nm and the sphere circumference 2*πρ*. The sphere circumference serves as the reference length-scale for the diffusion time-scale *τ*_*R*_. We perform stochastic simulations using these parameters with a time step at most Δ*t* = *τ*_*R*_/500.

We consider the case when the acceptor and donor are free to rotate but are held at a fixed separation distance *R*. We take *R* = *R*_0_ so that for perfect averaging over all of the orientation configurations the transfer efficiency is *E* = 0.5. We consider the rotational dynamics relative to the donor excitation life-time characterized by *τ*_*D*_/*τ*_*R*_.

**Fig 4:**
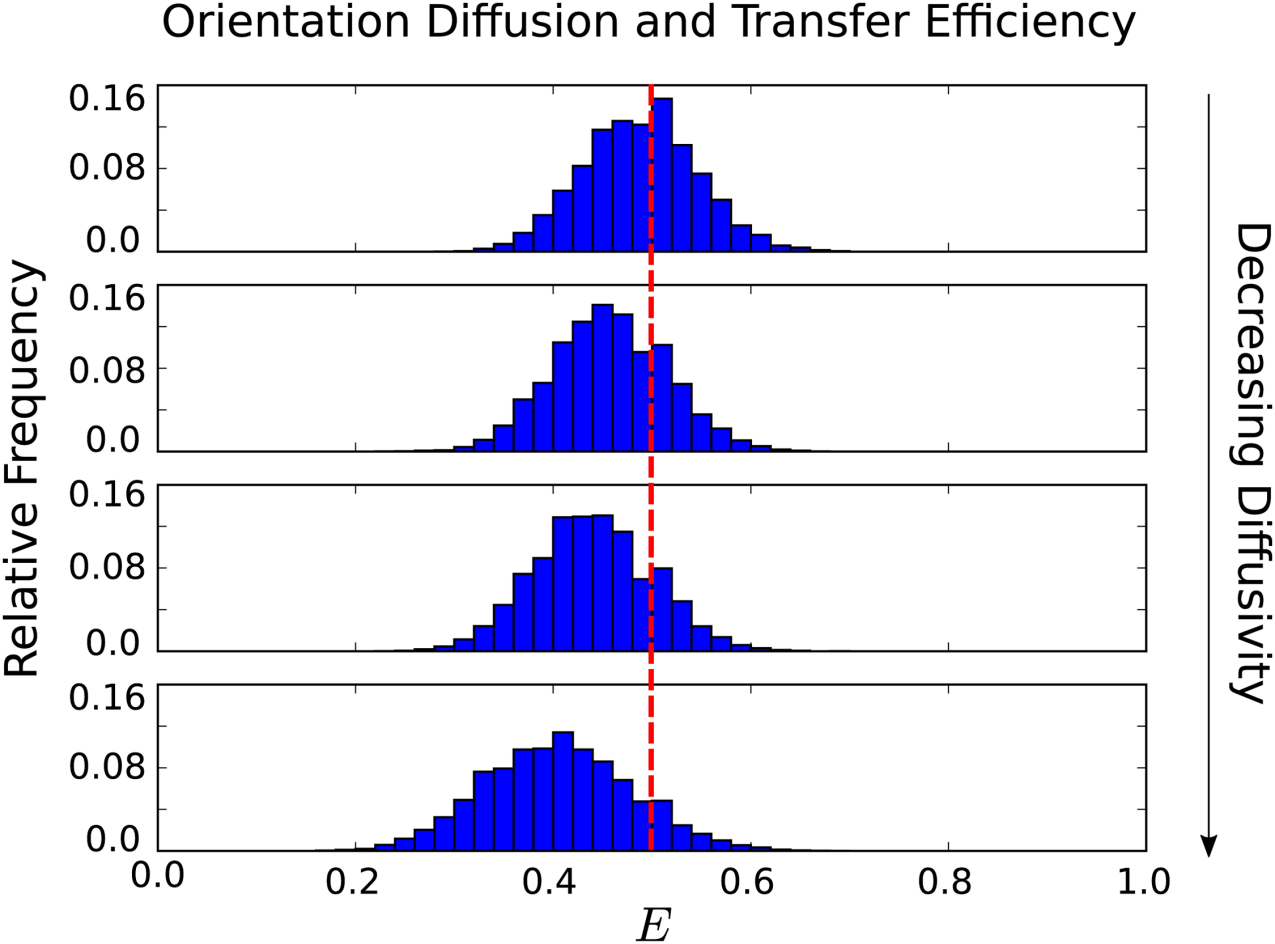
Rotational diffusion and shifts in FRET transfer efficiency *E*. From top to bottom are dyes with decreasing rotational diffusion having characteristic diffusion times *τ*_*R*_/*τ*_*D*_ = 19.5, 97.5, 195.0, 975. The average efficiencies in each case are respectively *E* = 0.486, *E* = 0.456, *E* = 0.438, and *E* = 0.403. The shift in average efficiency from the slowest to fastest diffusion considered is about 20%. A notable feature for decreasing diffusivity is that the distribution of observed efficiencies broadens. The reference efficiency *E*_0_ = 0.5 is indicated by the red line.

We consider both fast rotational diffusion where most configurations are well-sampled over the donor lifetime *τ*_*D*_/*τ*_*R*_ ≫ 1, and slow rotational diffusion where only a very limited subset of configurations are sampled over the donor lifetime *τ*_*D*_/*τ*_*R*_ ≪ 1. For slow rotational diffusion, we find that the limited sampling over the donor lifetime can result in significant shifts of the observed FRET transfer *E* toward lower efficiencies, see Fig 4.

**Fig 5:**
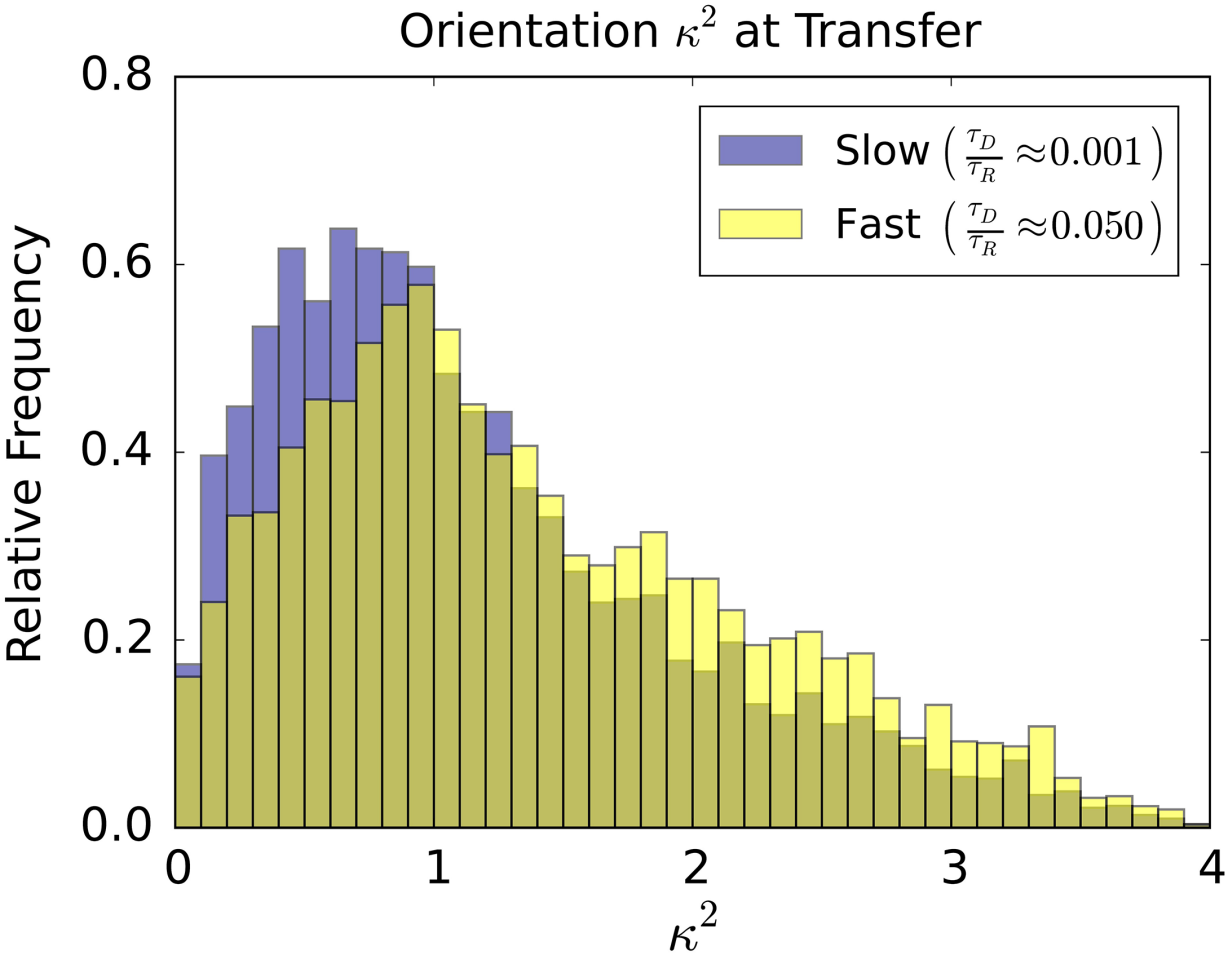
Orientation factor at time of transfer. Shown are the factors *κ*^2^ that occurred in the simulation at the time of energy transfer from the donor to the acceptor. We compare the case of slow rotational diffusion *τ*_*D*_/*τ*_*R*_ = 0.001 and fast rotational diffusion *τ*_*D*_/*τ*_*R*_ = 0.05. For the slow rotational diffusion *κ*^2^ factors exhibit a significant shift toward smaller values. This is a consequence of the fast rotational diffusion having more opportunities to be in favourable orientations for energy transfer.

The orientation configurations are all equally likely and the factor *κ*^2^ contributes linearly to the transfer efficiency in Eq (3). As a consequence, the shift exhibited is a result of purely kinetic effects. In particular, for the fastest rotational diffusion the donor and acceptor have more opportunities to occupy orientations that are favourable to energy transfer. In other words, when the diffusion is large the donor and acceptor have time to diffuse to encounter configurations that are in a “sweet spot” having the largest chance of triggering energy transfer. When the rotational diffusion is much slower than the donor lifetime, the donor and acceptor orientation remain close to the initial starting configuration which primarily determines the rate of energy transfer. This manifests as a shift in the *κ*^2^ values toward the smaller values corresponding to less efficient transfer when the rotational diffusion is slow relative to the donor lifetime, see Fig 5.

The shift in transfer efficiencies resulting from the rotational kinetics can be significant. For a relatively fast rotational diffusion on the time-scale *τ*_*R*_/*τ*_*D*_ = 19.5, we find the energy transfer is *E* = 0.486. This is close to when orientation is fully averaged to yield the energy transfer *E* = 0.5. For a slow rotational diffusion time-scale of *τ*_*R*_/*τ*_*D*_ = 975 we have a transfer efficiency of *E* = 0.403. In this case, the rotational kinetics has resulted in a shift in the average transfer efficiency of 17%.

**Fig 6:**
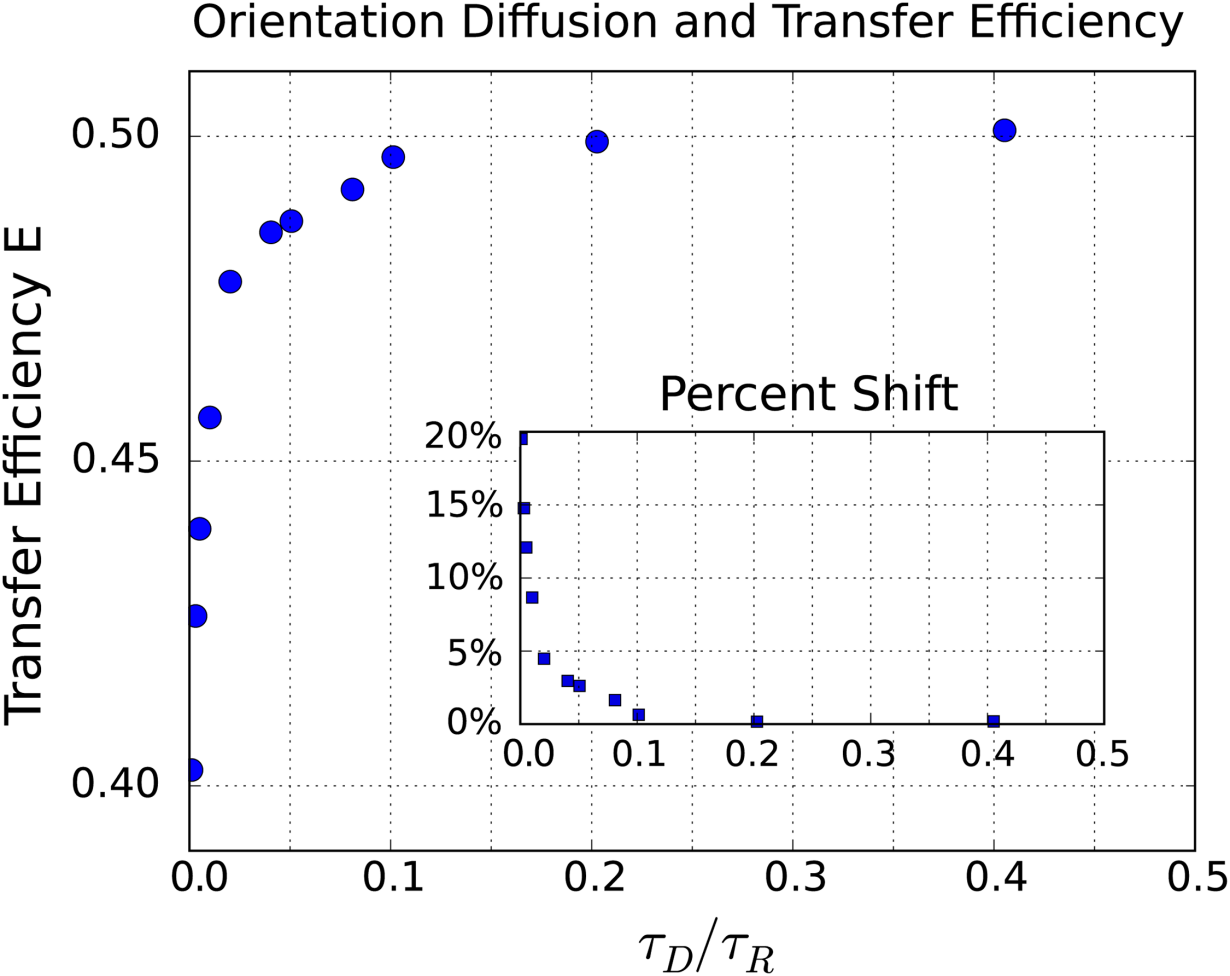
Rotational diffusion and shifts in FRET transfer efficiency *E*. As the rotational diffusion decreases the mean transfer efficiency shifts significantly. In the inset, we show the percentage shift measured as **% shift** = |*E*_obs_ − *E*_0_|/*E*_0_ where we take the reference efficiency *E*_0_ = 0.5. The first few data points have *τ*_*D*_/*τ*_*R*_ = 0.001, 0.003, and 0.005.

Our results indicate on way that the FRET transfer efficiency *E* can exhibit a significant shift without any change in the conformational state of the measured molecule. These changes arise purely from different rates of rotational diffusion. In practice, this could arise from changes in the viscosity of the surrounding solvent or from transient binding events with molecules present in the solvent that transiently restrict rotation of the donor and acceptor. We show over a range of diffusivities the shifts that can occur from these effects in Fig 6.

### 3.3 Donor-acceptor diffusion in separation distance

We consider the role of the relative translational diffusion of the donor and acceptor molecules. We are particularly interested in the case when the measured molecule’s conformational state involves a sampling over an ensemble of different configurations. In this case, the donor and acceptor could undergo significant translational diffusion over the donor lifetime [51, 52]. For instance, for a disordered protein or a polymer subjected to different solvation conditions FRET could be used to get an indication of the radius of gyration [35, 53–55]. When the ensemble of configurations remains unchanged, we investigate the role of the kinetics associated with the diffusion of the separation distance.

We model the diffusion of the separation distance *R* by the stochastic process

$$\begin{array}{ccc} \frac{dR_{t}}{dt} & = & -\frac{1}{\gamma }\Phi^{\prime }\left(R_{t}\right)dt+\sqrt{2D_{S}}\frac{dW_{t}}{dt} \end{array}$$ (10)

The *γ* denotes the effective drag, Φ the potential of free energy for the separation distance *R*, *D*_*S*_ the effective diffusivity in separation, and *W*_*t*_ Brownian motion. The equation is to be interepreted in the sense of Ito Calculus [48]. We model the separation of the donor and acceptor labels attached to the polymer by the potential of free energy

$$\begin{array}{c} \Phi \left(r\right)=\frac{k}{2}{\left(r-\ell \right)}^{2} \end{array}$$ (11)

We parameterize the model using the diffusivity *D*_*S*_ and take the drag *γ* = *k*_B_*T*/*D*_*S*_ where *k*_B_ is Boltzmann’s constant and *T* is temperature. To model what happens as the separation distance approaches zero, we avoid negative lengths by a reflecting boundary condition at zero [49]. We characterize this diffusive dynamics by the time-scale *τ*_*S*_ = *ℓ*^2^/*D*_*S*_ where *ℓ* is the same length that appears in Eq (11). The parameters used by default in our simulations are given in Table 1.

**Table 1:** Parameter values for the simulations.

Parameter	Value
*R*_0_	5.4*nm*
*τ*_*D*_	4*ns*
*k*_*B*_*T*	4.1 × 10^−21^ *J*
*k*	0.25*k*_*B*_*T*
*ℓ*	5.4*nm*

At equilibrium this diffusion process has the separation distribution

$$\begin{array}{c} \Psi \left(r\right)=\frac{1}{Z}\exp\left(-\Phi \left(r\right)/K_{B}T\right) \end{array}$$ (12)

where $Z=\int_{-\infty }^{\infty }\exp(-\Phi (|r|)/K_{B}T)dr$ is the partition function [56]. To simulate this process we generate time-steps using the Euler-Marayuma method [57]

$$\begin{array}{c} R^{n+1}=R^{n}-\frac{1}{\gamma }\Phi^{\prime }\left(R^{n}\right)\Delta t+\sqrt{2D_{S}\Delta t}\eta^{n} \end{array}$$ (13)

**Fig 7:**
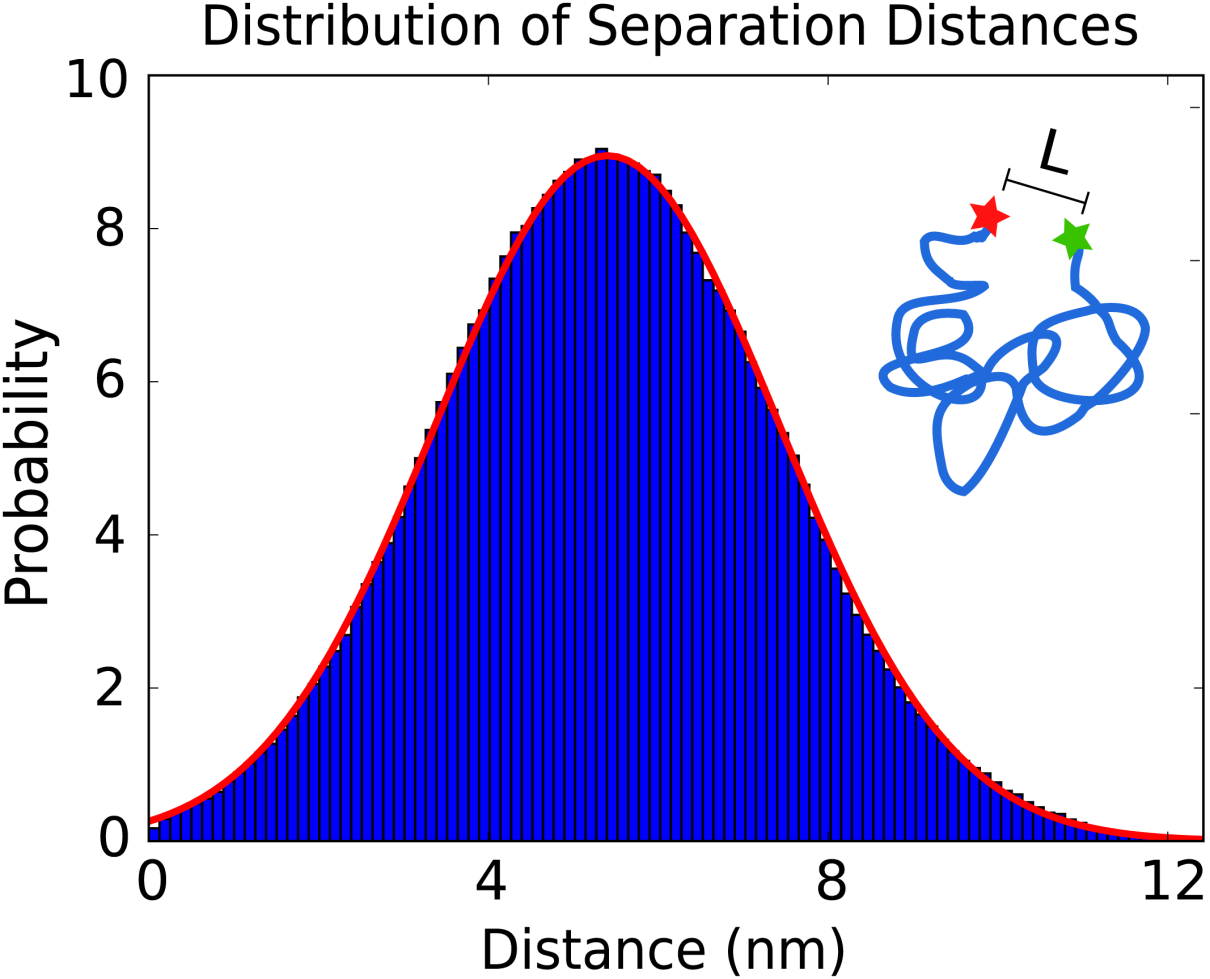
Equilibrium distribution of donor-acceptor separation distances. The results of simulated steps of the acceptor-donor labels of the polymer diffusion (histogram) are compared with the predicted distribution of separation distances from Eq (12) (red-curve). Results are obtained from 1.8 × 10^6^ sampled simulation steps fit with mean *μ* = *R*_0_ and variance $\sigma^{2}=0.14R_{0}^{2}$.

The *η*^*n*^ is generated each time-step as an independent standard Gaussian random variable with mean zero and variance one. The time-step duration is denoted by Δ*t*. In practice, we use a time-step with Δ*t* = *τ*_*S*_/10^4^. To give some intuition for the separation fluctuations and as a validation of our simulation methods, we show numerical results for the equilibrium distribution in Fig 7.

We consider the role of the separation kinetics of the donor and acceptor over the donor excitation life-time. We consider the transfer efficiency for different rates of separation diffusion *D*_*S*_ relative to the donor life-time *τ*_*D*_. This can be characterized by *τ*_*D*_/*τ*_*S*_ where *τ*_*S*_ = *ℓ*^2^/*D*_*S*_.

**Fig 8:**
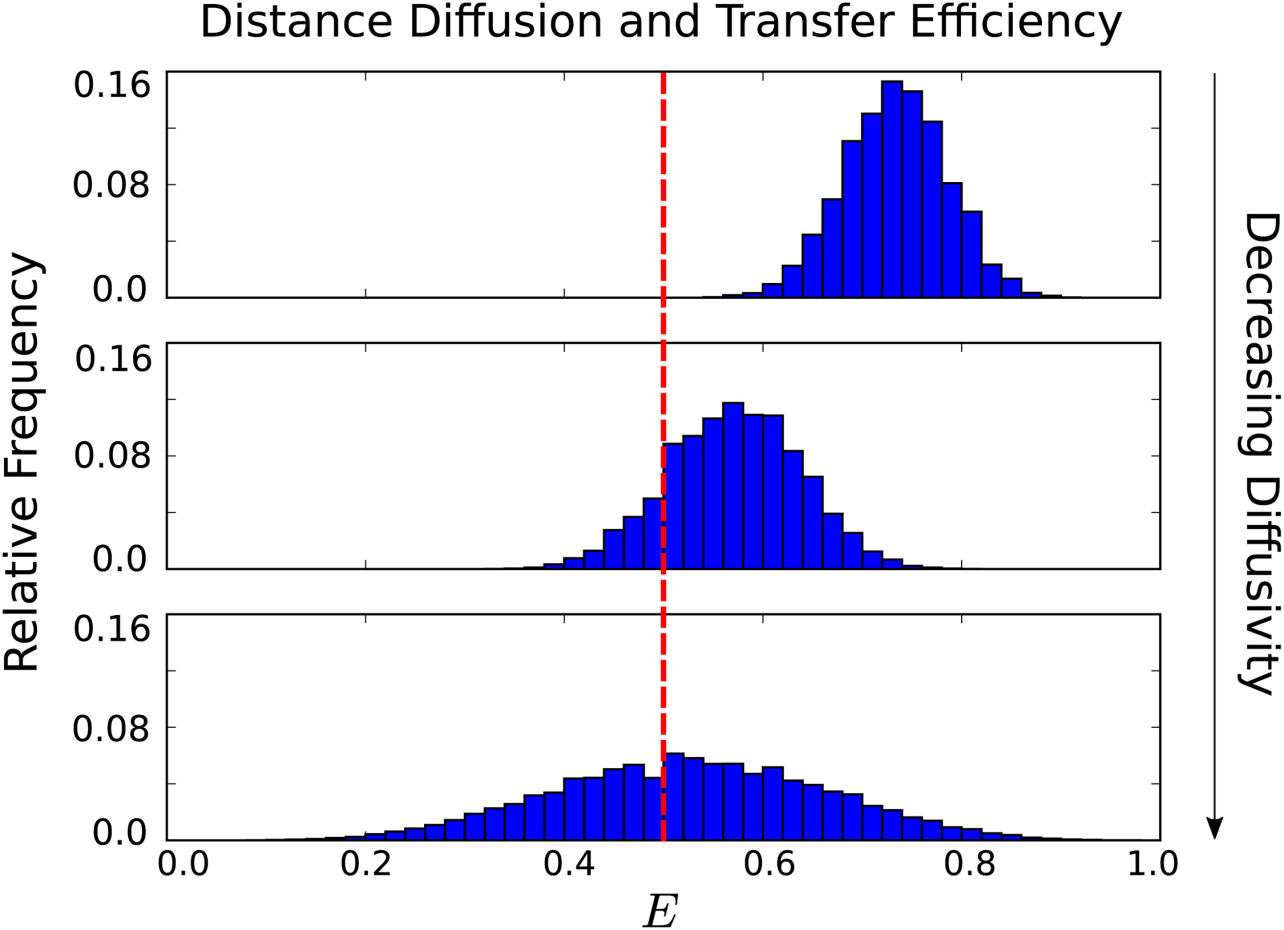
Separation diffusivity and FRET transfer efficiency. The separation diffusivities correspond to *τ*_*D*_/*τ*_*S*_ = 0.69, 0.07, and 0.007. These have mean transfer efficiencies respectively *E* = 0.723, *E* = 0.553, and *E* = 0.508. This represents a relative shift of 30% in the transfer efficiency. As the separation diffusivity decreases the distribution of transfer efficiencies significantly broadens.

We find that a decrease in the separation diffusivity results in a significant shift in the FRET transfer efficiency, see Fig 8. We also find that as the separation diffusivity decreases the distribution of observed efficiencies broadens significantly. For the fastest translational diffusivity we have a mean transfer efficiency of *E* = 0.723 verses for the slowest translational diffusivity considered *E* = 0.508. This gives a relative shift in the FRET transfer efficiency of 30%.

The ensemble of configurations is the same for both the fastest and the slowest diffusion so the shift in transfer efficiency arises purely from kinetic effects. Over the donor life-time, the diffusion influences how likely the donor and acceptor are to encounter configurations favourable to energy transfer. In the case of slow diffusion, the rate of energy transfer is primarily governed by the initial configuration of the donor and acceptor.

**Fig 9:**
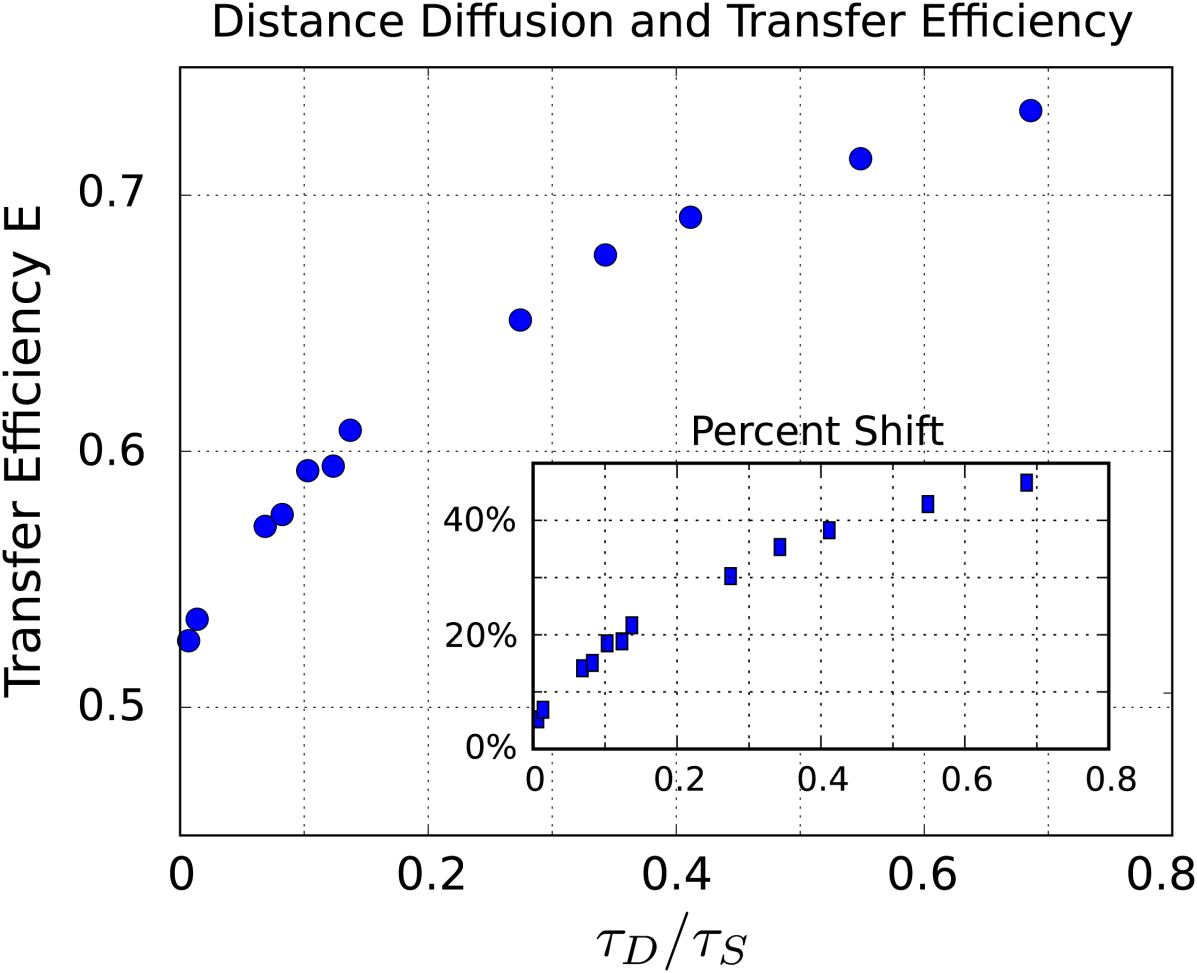
Separation distance at time of energy transfer. Shown are the separation distances that occurred in the simulation at the time of energy transfer. We compare the case of slow distance diffusion *τ*_*D*_/*τ*_*S*_ = 0.007 and fast distance diffusion *τ*_*D*_/*τ*_*S*_ = 0.69. For the case of fast diffusion we see that the energy transfer occurs much more frequently at shorter separation distances.

In the case of fast diffusion relative to the donor life-time, the donor and acceptor have more of an opportunity to encounter favorable configurations for energy transfer. This difference in how often such “sweet spots” for energy transfer are encountered over the donor life-time is supported by the observed separation distances that occur at the time of energy transfer, see Fig 9.

**Fig 10:**
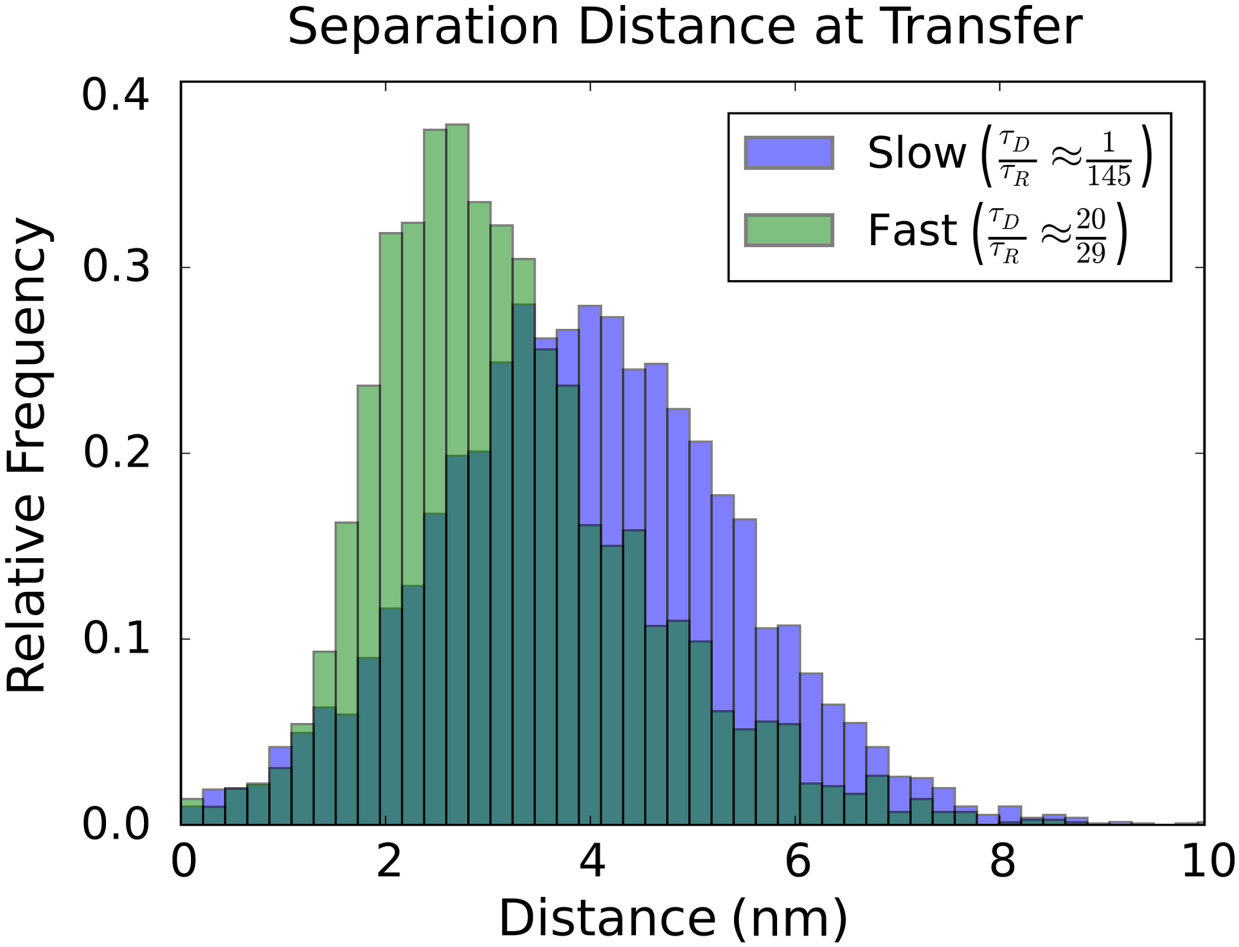
Distance diffusion and shifts in FRET transfer efficiency. As the distance diffusion decreases the mean transfer efficiency shifts significantly. In the inset, we show the shift as a relative percentage given by **% shift** = |*E*_obs_ − *E*_0_|/*E*_0_ with reference efficiency *E*_0_ = 0.5.

For the fastest diffusivity, we see that significantly smaller separation distances occur at the time of energy transfer and thus yield on average larger FRET efficiencies. In the case of the slowest diffusivity, we see that the distribution of separation distances is broader and more closely follows the equilibrium distribution of separation distances since the rate of energy transfer is largely determined by the initial configuration of the donor and acceptor. We show the shifts in energy transfer for a wide range of separation diffusivities in Fig 10.

### 3.4 Role of non-emitting quenching

We also consider the case when the donor can de-excite through a non-emitting pathway [29]. One possible mechanism is dynamic quenching where the donor de-excites by making contact with chemical species diffusing in the surrounding solution [26–28]. Some donors have photo-physics that are significantly impacted by the presence of ions. This is used in some experiments as a reporter on ion concentration [15, 26, 31].

We take these effects into account by developing some theory for how an additional non-emitting pathway would shift the observed FRET efficiency. A non-emitting quenching pathway can be modelled in our kinetics by killing some fraction of the donor de-excitation events that would have resulted in energy transfer to the acceptor and ultimately emission of acceptor photons. For the FRET transfer efficiency this corresponds to augmenting Eq (5) to

$$\begin{array}{c} \tilde{E}\left(\alpha \right)=\frac{\alpha n_{A}}{n_{D}+\alpha n_{A}} \end{array}$$ (14)

The quantity 1 − *α* gives the fraction of donor de-excitations that result in some type of non-emitting quencher event. The $\tilde{E}\left(\alpha \right)$ gives the corresponding shifted FRET efficiency when including the quenching pathway.

**Fig 11:**
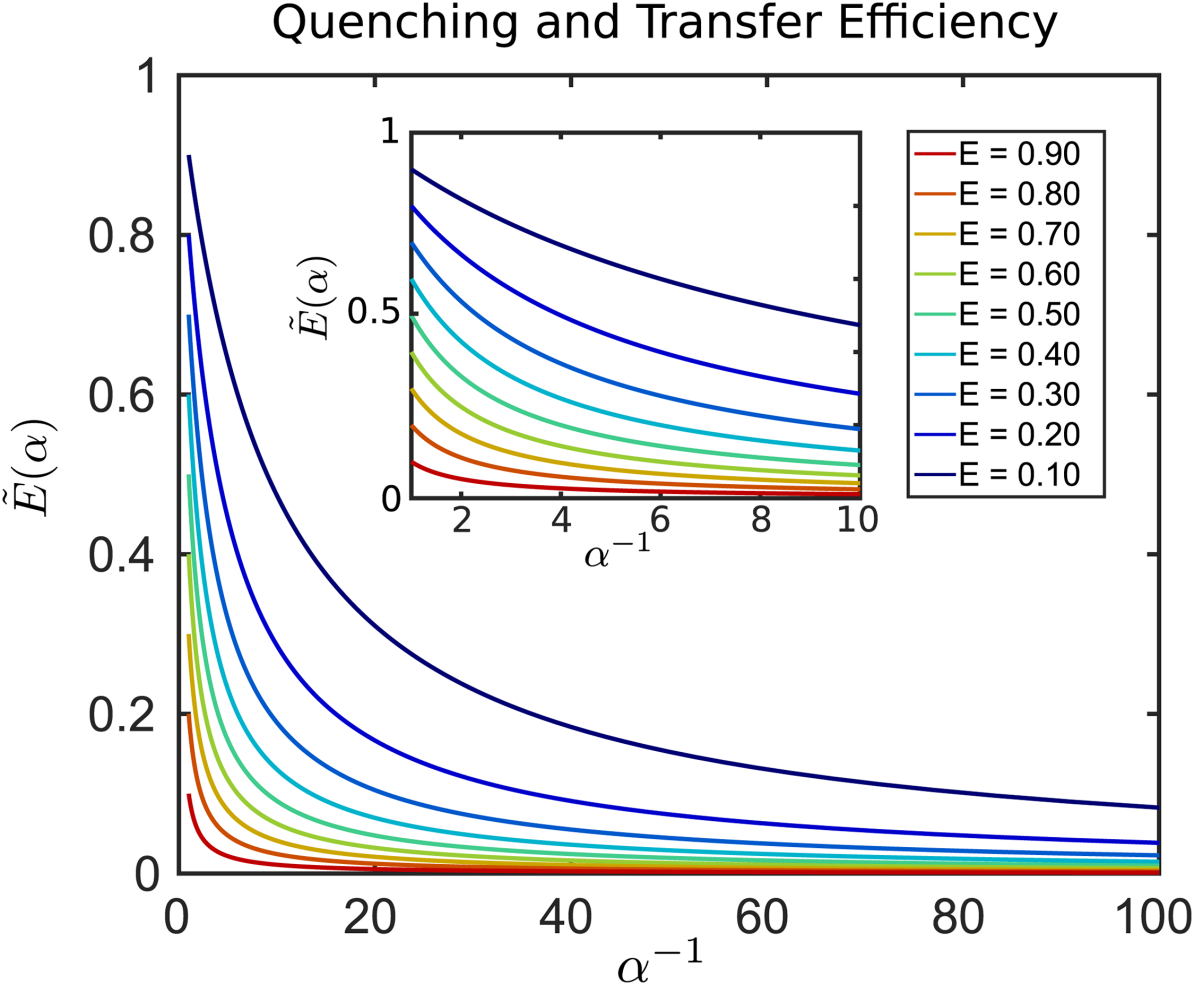
Non-emitting quenching and shifts in FRET transfer efficiency. The observed FRET transfer efficiency $\tilde{E}\left(\alpha \right)$ is shown when incorporating an additional non-emitting pathway in the donor-acceptor kinetics. For different rates *α* of non-emitting quenching events, the results show how a reference transfer efficiency *E* in the case of no quenching is augmented.

The case *α* = 1 corresponds to the situation when no non-emitting quenching events occur. In this case we have $\tilde{E}\left(1\right)=E$. In the case of *α* = 0, all observed de-excitations result in non-emitting quencher events instead of donor de-excitation through FRET transfer events and emission of acceptor photons. In this case we have $\tilde{E}\left(0\right)=0$, see Fig 11.

The FRET efficiency can be conveniently expressed as

$$\begin{array}{c} \tilde{E}=\frac{1}{\alpha^{-1}f+1} \end{array}$$ (15)

where $f=(\frac{n_{D}}{n_{A}})$. This provides a reference *f* corresponding to the ratio of donor to acceptor emissions when there is no non-emitting quenching. The reference fraction *f* is related to a reference FRET transfer efficiency *E* by *f* = *E*^−1^ − 1. The percentage shift of the observed FRET efficiency that arises from quenching is given by

$$\begin{array}{c} s=\frac{E-\tilde{E}\left(\alpha \right)}{E}=1-\frac{1}{\alpha^{-1}\left(1-E\right)+E} \end{array}$$ (16)

**Fig 12:**
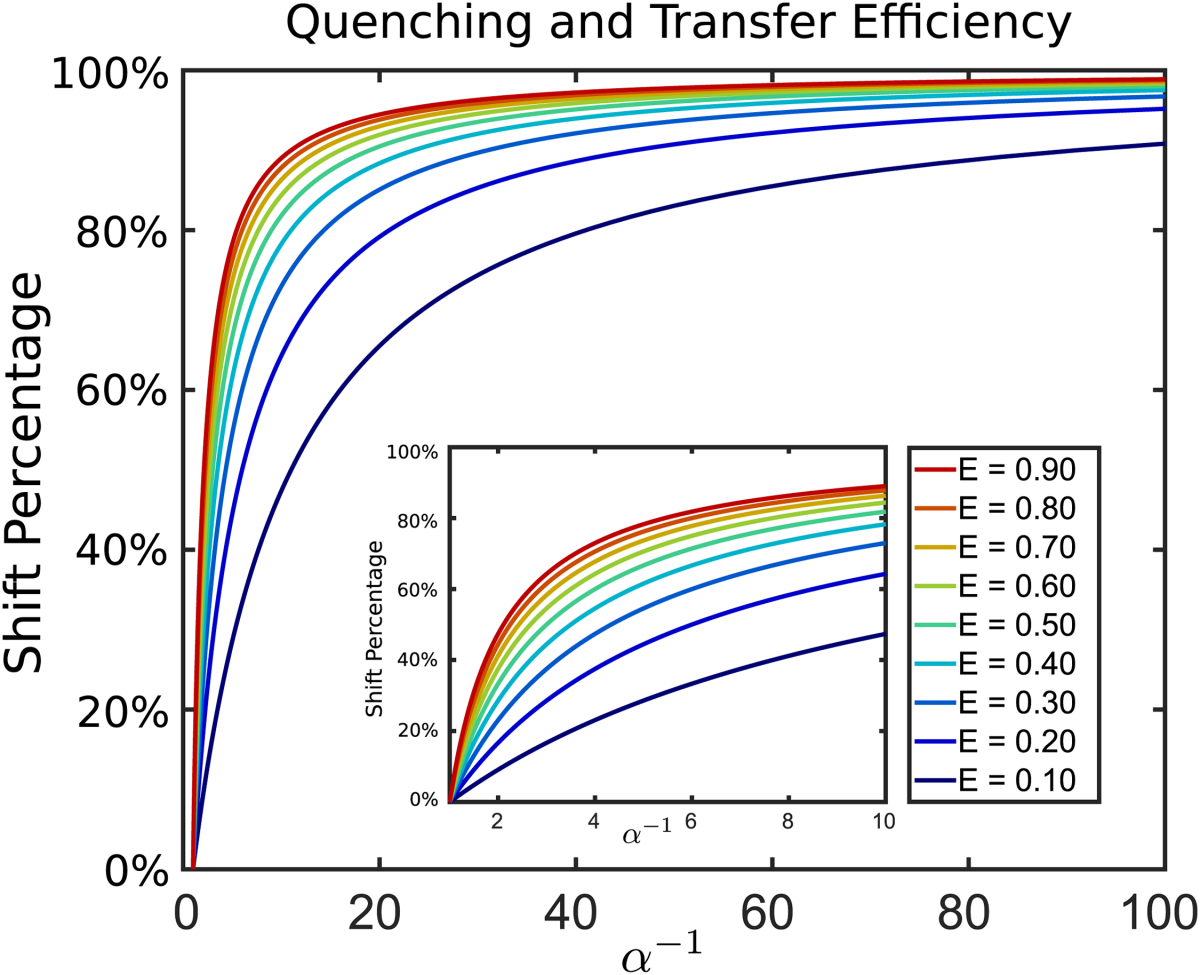
Non-emitting quenching and shifts in FRET transfer efficiency. The relative percentage shift $s=E-\tilde{E}\left(\alpha \right)/E$ in transfer efficiency is shown when non-emitting quenching occurs as part of the donor-acceptor kinetics.

We see that the percentage shift in FRET that occurs from quenching has a dependence on the reference FRET transfer efficiency *E*. In fact, the shift that occurs becomes increasingly sensitive as *E* decreases, see Fig 12.

## 4 Discussion

We have shown a few different ways that FRET efficiency can be shifted as a consequence of kinetic effects while the underlying molecular conformational state in fact has remained the same. We consider how such kinetic mechanisms relate to some recent experiments investigating the origins of shifts in FRET efficiency [19, 34, 35, 58].

FRET is often used to measure conformational changes or folding of proteins as denaturant conditions are varied [22, 35, 53]. In the recent work by Lipman, Plaxco, et al [35], the radius of gyration of polyethylene glycol (PEG) polymers are considered in solvation conditions that yield random-coils. Unlike proteins, the ensemble of PEG polymer configurations is not expected to change significantly when varying the denaturant. This is substantiated in the experiments by x-ray scattering measurements that show indeed the PEG radius of gyration remains unchanged when varying the denaturant [35, 58]. This provides a useful control to investigate FRET as the denaturant conditions are varied.

An interesting finding is that FRET measurements under the same conditions exhibit a significant shift in the measured transfer efficiency. For a 3*kDa* PEG polymer in denaturant *GuHcl* ranging in concentration from 0 − 6M molar a shift was observed in the transfer efficiency of ∼20% referenced from *E*_0_ = 0.5. For the same polymer in the denaturant *urea* ranging in concentration from 0 − 8M a shift was observed in efficiency of ∼24% referenced from *E*_0_ = 0.5. Similar shifts were found for experiments performed using 5*kDa* PEG [35].

Our results show that significant shifts can occur in the observed FRET efficiency even when there is no underlying change in the conformational ensemble. We showed how the transfer efficiency can shift purely from kinetic effects arising from changes in the rate of diffusion of the acceptor-donor orientation, diffusion of the separation distance between the donor and acceptor, and from non-emitting quenching. For diffusion of the donor-acceptor separation distance, we found such kinetic effects can cause shifts in efficiency as large as 48%. This occurred as the distance diffusion time-scale approached that of the donor life-time, see Fig 10.

One way to try to account for the experimentally observed shifts is to consider how the denaturant augments the viscosity of the solvent [35, 59]. Changes in the solvent viscosity are expected to be closely related to changes in the rate of diffusion as suggested by the Stokes-Einstein relation [49]. Such a mechanism was explored theoretically in the work [58, 60]. We discuss here how our simulation results relate to changes in the solvent viscosity.

The purported change in bulk solvent viscosity under changes in the *urea* denaturant concentration at 8M is the factor 1.66 and for *GuHcl* 6M the factor 1.61 according to the experiments in [59]. To relate viscosity to diffusivity, the Stokes-Einstein relation can be used *D* = *k*_*B*_*T*/*γ*. The drag is given by *γ* = 6*πμa* where *μ* is the solvent viscosity and *a* is a reference length-scale characterizing the size of the diffusing molecule. This suggests that by increasing the solvent viscosity by a factor of 1.61 reduces the diffusivity by a factor of 0.6.

In our simulations taking as the base-line case *τ*_*D*_/*τ*_*S*_ = 0.1, such a change in viscosity shifts the transfer efficiency by ∼12%. This contribution solely from the diffusive kinetics of the donor-acceptor separation accounts for about half the ∼24% shift observed for 8M *urea* and the ∼20% observed for 6M *GuHcl* in [35]. This is consistent with the findings in [58] suggesting other mechanisms may also play a role in the observed shift in transfer efficiency.

There are a number of potential subtleties when interpreting these effects. For one the donor and acceptor molecules are comparable in size to the viscogen denaturant molecules and the changes in diffusivity could possibly be more significant owing to more complicated interactions than suggested by the use of simple bulk theory for viscosity and diffusion [61–63]. Another consideration is the role played by non-emitting quenching caused by collisional contact of the denaturant molecules with the donor [29]. Combined with the kinetic changes in diffusion, even a modest amount of excitations resulting in quenching events <5% would lead to an overall combined shift of ∼20% in the observed transfer efficiency, see Fig 12.

## 5 Conclusion

We have shown that kinetics can play a significant role in shifting the observed FRET transfer efficiency even when there is no underlying change in the conformational state of the molecule being measured. We found that changes in the orientation diffusion can in the most extreme cases shift the transfer efficiency by up to 20%. For the considered diffusion of the donor-acceptor separation distance, we found in the most extreme cases shifts up to 48%. Our findings concerning the donor-acceptor distance are in agreement with the investigations of Makarov and Plaxco’s work [38]. We mention that our results concerning the orientation diffusion accounts for additional effects not in [38] and could offer some explaination of the FRET shifts that are seen in rigid polyproline chains [34, 35]. We found that the diffusive kinetics of both orientation and separation exhibit a distinct signature in the histogram of observed transfer efficiencies as a broadening of the peaks. We also found that non-emitting quenching events that occur even at a modest level can result in significant shifts in the observed transfer efficiency. The mechanisms we have discussed have potentially important implications when interpreting FRET measurements, especially with respect to making inferences from changes in the FRET distance and how this is attributed to changes in the conformational state of molecules. In analysing FRET measurements, we hope our results provide a few useful benchmarks to help determine the significance of observed shifts and the role of kinetic effects.
